# Current status of proximal gastrectomy for gastric and esophagogastric junctional cancer: A review

**DOI:** 10.1002/ags3.12365

**Published:** 2020-06-21

**Authors:** Souya Nunobe, Satoshi Ida

**Affiliations:** ^1^ Department of Gastroenterological surgery Cancer Institute Ariake Hospital Tokyo Japan

**Keywords:** gastric cancer, proximal gastrectomy, reconstruction method

## Abstract

Proximal gastrectomy (PG) is one of the function‐preserving surgical methods for the treatment of upper gastric cancer. Favorable postoperative results have been reported in comparison with total gastrectomy. However, because there are challenges, such as postoperative reflux esophagitis, anastomotic stenosis, and residual food, appropriate selection of a reconstruction method is crucial. Some methods include esophagogastric anastomosis, including simple esophagogastrostomy, tube‐like stomach esophagogastrostomy, side overlap with fundoplication by Yamashita, and double‐flap technique, and reconstruction using the small intestine, including double‐tract methods, jejunal interposition, and jejunal pouch interposition. However, standard reconstruction methods are yet to be established. PG has also been employed in early gastric cancer of the upper third of the stomach, and indications have also been extended to esophagogastric junction cancer, which has shown an increase in recent years. Although many retrospective studies have revealed the functional benefits or oncological safety of PG, the characteristics of each surgical procedure should be understood so that an appropriate reconstruction method, with a reflux prevention mechanism and minimal postoperative injury, can be selected.

## 1 INTRODUCTION

According to the Japanese Gastric Cancer Treatment Guidelines, standard gastrectomy is defined as the resection of at least two‐thirds of the stomach, including D2 lymph node dissection.^1^ Furthermore, total gastrectomy (TG) is usually indicated for upper gastric cancer. Conversely, function‐preserving gastrectomy (FPG), whereby gastric function is maintained to the detriment of the advantages that standard gastric cancer surgery provides, is performed to address the postoperative quality of life (QOL) of the patient. FPG, a procedure that preserves the esophagogastric junction (EGJ) and pylorus as well as the capacity of the remnant stomach to maintain a functional reservoir,^2^, ^3^ is not accurately defined in the guidelines. However, there are limited indications for proximal gastrectomy (PG), a representative FPG procedure, which is generally performed with curative intent in cases of early gastric cancer (EGC) of the upper stomach. A large retrospective study using the postgastrectomy syndrome assessment scale (PGSAS‐45) reported that PG reduces postgastrectomy symptoms more than TG.^4^ Furthermore, PG is expected to preserve the reservoir function of the remnant distal stomach, including the pyloric ring function that prevents duodenogastric reflux, and has been associated with a lower rate of dumping syndrome.^5^, ^6^, ^7^, ^8^

On the other hand, patients who undergo PG may suffer from heartburn or gastric fullness resulting in esophageal reflux, which could lead to a poor postoperative QOL.^9^, ^10^

Because there are no standard procedures, it is difficult to choose a reconstruction method after PG. These include double‐tract methods, jejunal interposition, and esophagogastric anastomosis (among others), and the choice must be made in consideration of the prevention of gastroesophageal reflux and ensuring a good dietary intake.

Proximal gastrectomy is also indicated for EGJ cancer, which has shown a recent increase in Japan. The Japanese Gastric Cancer Treatment Guidelines published the algorithm for the surgical treatment for EGJ cancer <4 cm in diameter, including early and advanced cancer, where the extent of the lymph node dissection falls within the range of PG.^1^ However, many high‐level lesions, such as EGJ, can make choosing the reconstruction method more difficult compared with typical cases of upper gastric cancer.

PG, which was mainly adapted for EGC patients, was shown to achieve favorable prognosis with reduced surgical invasiveness, and its indications overlap those of laparoscopic gastrectomy, a form of minimally invasive surgery.^11^, ^12^, ^13^ Additionally, due to the increased use of minimally invasive surgery, laparoscopic surgery has even been performed for advanced gastric cancer and advanced EGJ cancer. However, dissection of the distal pancreas and reconstruction of the esophageal hiatus is technically challenging when laparoscopic procedures are employed for PG.

In this review, we summarize the current landscape of the PG procedure, including indications, lymph node dissection, reconstruction methods, and remnant stomach cancer, for upper gastric and EGJ cancer.

## 2 INDICATIONS FOR PG

With the recently increasing incidence of proximal gastric cancer in Asian countries, PG is widely accepted as a FPG in EGC.^14^, ^15^, ^16^, ^17^ Some authors argue that PG is not oncologically and functionally preferred to TG for EGC located in the upper stomach. An analysis of outcomes in EGC patients treated using the standard Japanese D2 TG method in the 1980s showed that nodal metastasis to distal perigastric lymph nodes was rarely recognized^18^; therefore, dissection of these nodes was considered unnecessary. The 2018 Japanese Gastric Cancer Guidelines also recommended modified procedures, including PG, for the surgical treatment of cT1N0 gastric cancer.^1^ On the other hand, the standard procedure for advanced gastric cancer of the upper stomach should be TG, and PG is not considered standard. However, it was reported that distant side lymph node metastasis was rare if the tumor was localized to the upper stomach, making PG applicable for advanced gastric cancer of the upper stomach.^19^

The guidelines recommend that the size of the remnant stomach after PG should be half or more than the original size. Furthermore, because the size of the remnant stomach is closely related to the tumor location and the surgical margin, it is an indication itself for this procedure. Fundamentally, PG is a function‐preserving operation, and it is considered that the size and function of the residual stomach would be related. Nomura et al^20^ reported that in cases where the gastric remnant after distal gastrectomy was small, food intake was significantly decreased. Furthermore, if the remnant stomach volume cannot be maintained, the gastric emptying pattern was shown to worsen.^20^ We have also found that a residual stomach size <2/3 of the preoperative volume was an independent risk factor for skeletal muscle index reduction 1 year after PG because a smaller remnant stomach appeared to be associated with decreased food intake and the deterioration of peristalsis, subsequently resulting in skeletal muscle loss (data under submission). Therefore, perioperative nutritional interventions may be crucial in cases with an inadequate residual stomach volume.

Another interesting decision‐making question is whether to choose PG or subtotal gastrectomy for EGC of the upper stomach.^21^ Subtotal gastrectomy may be possible for lesser curvature lesions 3 cm from the EGJ. For lesions of the posterior wall or greater curvature, lymph node dissection on the distal side of the pancreas is also necessary, and this is often indicated for PG. Kano et al^22^ showed that in cT1 lesions of the upper stomach, few lymph nodes could be dissected unless PG, but subtotal resection showed a significantly shorter margin for resection. Either procedure may be used for early‐stage cancer, as long as the resection margin can be secured, but if the remaining stomach is preserved in cases of advanced gastric cancer of the upper stomach, PG may be performed due to an adequate surgical margin.

The guidelines defined an algorithm for the dissection range of early and advanced EGJ cancers of ≤4 cm. In this category, lymph node dissection on the pyloric side is unnecessary, and PG is theoretically possible. Large phase II studies of EGJ cancers deeper than T2 have shown similar results, regardless of tumor size.^23^

## 3 LYMPH NODE DISSECTION DURING PG

The Japanese Gastric Cancer Treatment Guideline defines PG as a function‐preserving operation for cT1N0 ECG of the upper stomach, with the extent of lymph node dissection as D1 or D1+. Lymph node dissection in the distal part of the pancreas is unnecessary in this adaption. If PG is indicated for advanced gastric cancer, D2 lymph node dissection will be defined, and lymph node dissection distal to the pancreas will be required. Yura et al^19^ investigated the dissection effect of each lymph node station based on the frequency of lymph node metastasis from advanced gastric cancer (pT3 or pT4) in the upper stomach, confirming that dissection of the lymph node in the distal part of the pancreas had the same effect as the dissection of the lymph node at the root of the celiac artery. Lymph node dissection distal to the pancreas may be necessary for advanced gastric cancer of the upper stomach when PG would be indicated. However, the intention of extent of lymphadenectomy or type of gastrectomy has to be decided based on the clinical stage, considering that the clinical and pathological stages did not coincide with each other like in previous reports.^24^, ^25^ Therefore, limited surgery to TG for upper advanced gastric cancer should be carefully applied.

First reported by Uyama et al in 1995, laparoscopic PG has been increasingly performed, and several technical reports and case studies with small sample sizes have been published.^11^, ^12^, ^13^, ^26^, ^27^ Lymph node dissection at the distal part of the pancreas is a difficult procedure in laparoscopic surgery, requiring surgical expertise. Thus, the use of PG as a standard procedure in laparoscopic surgery for advanced gastric cancer of the upper stomach will have to address not only the problem of the extent of the lymph node dissection but also the technical difficulties.

As described above, dissection of the lymph nodes on the pyloric side may become unnecessary in cases of EGJ cancer.^1^, ^23^ The results of a large phase II study suggested that lymph node dissection of the inferior mediastinum was required when the length of the esophageal invasion exceeded 2 cm.^23^

## 4 RECONSTRUCTION AFTER PG

There are various postgastrectomy reconstruction methods, and the method chosen must prevent reflux esophagitis and ensure a good dietary intake. As well as the short‐term results of the reconstruction method, the extent of long‐term nutritional effects, state of anemia, and frequency of gastric cancer in the remnant stomach are important points to consider.

There are two major methods of reconstruction after PG; one uses esophagogastric anastomosis, and one uses the small intestine. Esophagogastric anastomosis includes simple esophagogastrostomy, tube‐like stomach esophagogastrostomy, side overlap with fundoplication by Yamashita (SOFY), and the double‐flap technique. Reconstruction methods using the small intestine include the double‐tract, jejunal interposition, and jejunal pouch interposition methods. Because EGJ cancer requires a higher level of anastomosis than typical gastric cancer, a more careful choice of reconstruction is essential.

In particular, a high level of anastomosis would be required depending on the advanced stage of EGJ cancers. Reconstruction with a gastric tube is stable for high level of anastomosis, but reflux is a complication after surgery. Although there are restrictions on raising the small intestinal mesentery, it would be better to select this method if safe reconstruction is possible. Kurokawa et al conducted a large‐scale phase II study of EGJ cancer deeper than T2, in which 180 (49.6%) of 363 enrolled cases underwent PG.^23^ If detailed results are published, the reconstruction method preferred by many surgeons and its safety will be known.

In recent years, PG by laparoscopic procedure has been widely used; however, reconstruction may be a major technical complication. A prospective phase II study (JCOG1401) was conducted to confirm the safety of esophagogastric anastomosis or esophagojejunostomy after TG or PG. This study reported that esophageal anastomotic failure was 2.5% and confirmed the safety of laparoscopic reconstruction.^28^ In this study, PG was performed in 49 (20%) of 244 cases, and the double‐tract method was performed in 45 cases (92%) and jejunal interposition in four cases (8%) as reconstruction methods after PG. The results of this study suggest that the double‐tract methods tend to be favored in laparoscopic PG.

The exclusion criteria of the JCOG1401 study included “no esophageal invasion.” Therefore, there was no clear evidence regarding the safety of laparoscopic PG for EGJ cancer. However, many institutes have extensively performed laparoscopic PG for EGJ cancer, possibly favoring the stability of lymph node dissection by a trans‐hiatal approach.

However, determining which reconstruction method after PG would be the best is challenging. Another PGSAS‐45 study is currently underway on the reconstruction method after PG for upper gastric cancer. At present, there is no standard reconstruction method after PG; however, when the results of this research are obtained, a guideline for a reconstruction method with high QOL could be obtained. It would be possible to perform randomized controlled trials if the facility is accustomed to the surgical methods with respect to the high level of reconstruction methods from the results of this study.

We describe some common methods of reconstruction following PG below. Tables 1 and 2 summarize the reports on the reconstruction methods after PG.

**Table 1:** Characteristics of studies which reported the reconstruction after proximal gastrectomy

Reference	Number of patients	Design	Types of reconstruction
Sakuramoto et al^27^	26	rCS	EG
Seshimo et al^30^	64	rCS	EG vs JI
Masuzawa et al^31^	81	rCS	EG vs JI
Tokunaga et al^32^	76	rCS	EG vs JI
Chen et al^33^	34	rCS	EG
Chen et al^34^	76	rCS	EG vs tEG
Hoshikawa et al^35^	41	rCS	EG vs JPI
Nakamura et al^36^	101	rCS	EG vs JI vs JPI
Ahn et al^13^	50	rCS	EG
Hosogi et al^37^	15	rCS	tEG
Mochiki et al ^38^	41	rCS	tEG
Adachi et al^39^	30	rCS	tEG vs JI
Yamashita et al^40^	14	rCS	SOFY
Kuroda et al^7^	33	rCS	DFT
Hayami et al^8^	43	rCS	DFT
Hosoda et al^42^	40	rCS	DFT
Muraoka et al^43^	24	rCS	DFT
Ahn et al^45^	43	rCS	DT
Katai et al^5^	128	rCS	JI
Takagawa et al^47^	38	RCT	JI vs JPI
Shinohara et al^48^	18	rCS	JI
Yabusaki et al^49^	159	rCS	JI vs JPI
Kinoshita et al^6^	90	rCS	JI
Nozaki et al^50^	102	rCS	JI
Namikawa et al^51^	22	rCS	JPI
Yoo et al^52^	25	pCS	JPI

Abbreviations: DFT, double flap technique; DT,, double tract; EG,, esophagogastrostomy; JI, jejunal interposition; JPI,, jejunal pouch interposition; pCS, prospective case series; rCS, retrospective case series; RCT,, randomized controlled trial; SOFY,, side overlap with fundplication by Yamashita; tEG, tube‐like esophagogastrostomy.

**Table 2:** Surgical outcomes of reconstructions after proximal gastrectomy

Types of reconstruction	Morbidity	Stenosis	Reflux esophagitis
Esophagogastric anastomosis			
Esophagogastrostomy^13^, ^27^, ^30^, ^31^, ^32^, ^33^, ^34^, ^35^, ^36^	3.1%‐24%	0%‐52.2%	20%‐65.2%,
Tube‐like stomach esophagogastrostomy^34^, ^37^, ^38^, ^39^	0%‐20%	7.1%‐20%	5.7%‐30.8%
SOFY^40^	0%	0%	7.1%
DFT^7^, ^8^, ^42^, ^43^	3.0%‐25%	4.7%‐29.1%	0%‐8.3%
Reconstruction using the small intestinee			
Double tract ^45^	11.60%	4.70%	4.70%
Jejunal interposition ^5^, ^6^, ^30^, ^31^, ^32^, ^36^, ^39^, ^47^, ^48^, ^49^, ^50^	0%‐31.6%	3.1%‐31.8%	0%‐33.3%,
Jejunal pouch interposition ^35^, ^36^, ^47^, ^49^, ^51^, ^52^	3.6%‐25%	0%‐27.8%	4%‐27.8%

Abbreviations: DFT, Double flap technique; SOFY, side overlap with fundplication by Yamashita.

### 4.1 Esophagogastrostomy

Esophagogastrostomy, also called simple esophagogastrostomy, was used as the reconstruction method after Mikulicz's first PG in 1897.^29^ Esophagogastrostomy after PG is the simplest and most convenient physiological reconstruction method. However, without additional anti‐reflux treatment, the rate of reflux esophagitis is high after surgery, which greatly impairs the postoperative QOL. Additionally, this reconstruction method results in a high rate of stenosis of the anastomosis due to scarring and inflammation caused by reflux, which can lead to decreased dietary intake and worsened nutritional status. Several retrospective studies of esophagogastrostomy have observed early complications, stenosis, reflux esophagitis, and residual food in 3.1%‐24%, 0%‐52.2%, 20%‐65.2%, and 21.8% of cases, respectively. The most commonly observed complications were reflux esophagitis and residual food.^13^, ^27^, ^30^, ^31^, ^32^, ^33^, ^34^, ^35^, ^36^

### 4.2 Tube‐like stomach esophagogastrostomy

The postoperative condition of patients who underwent a tube‐like stomach esophagogastrostomy has been reported in several studies.^34^, ^37^, ^38^, ^39^ The incidence of patients who developed morbidity, stenosis, and reflux esophagitis was 0%‐20%, 7.1%‐20%, and 5.7%‐30.8%, respectively. Although no data on food residues for this procedure were found, stenosis was of interest.

### 4.3 SOFY method

Side overlap with fundoplication by Yamashita is a recently developed method of esophagogastric anastomosis with a unique anti‐reflux mechanism and can be easily performed laparoscopically.^40^ In this procedure, a linear stapler is used to create a slit‐shaped anastomosis using side overlap anastomosis in the anterior wall of the residual stomach to serve as a backflow prevention mechanism. In the preliminary report, 13/14 patients who received SOFY were asymptomatic without a proton pump inhibitor. This promising technique may become more widespread if positive long‐term surgical results are obtained.

### 4.4 Double‐flap technique

The double‐flap technique involves esophagogastric anastomosis after PG to which a fundoplication based on valvuloplasty was added by Kamikawa et al^41^ in 2001. Although this excellent reconstruction method prevents reflux and enables the intake of smooth meals, it has drawbacks of a flap formation step and complications, such as performing anastomosis by hand sewing. Hayami et al^7^ had favorable outcomes, including morbidity and nutritional status, after laparoscopic PG with the double‐flap technique compared with laparoscopic TG. Earlier reports found that the frequency of stenosis and rarity of reflux were 4.7%‐29.1% and 0%‐8.3%, respectively, and that caution was required when performing this procedure.^7^, ^8^, ^42^, ^43^ Shoji et al^44^ reported that stenosis was significantly increased, with an esophageal diameter of 18 mm as the cut‐off measure at the level of the diaphragm crus in preoperative computed tomography.

Although the double‐flap technique after PG is an excellent reconstruction method, the complications associated with the laparoscopic suturing technique must be solved for its widespread use. Stress‐free laparoscopic suturing, which is indispensable for this surgical procedure, may be performed using robotic surgery, which has become increasingly popular in recent years.

### 4.5 Double‐tract method

A retrospective study reported the results of surgical outcomes after using the double‐tract method for PG.^45^ Morbidity, stenosis, reflux syndromes, dumping syndrome, and residual food were reported in 11.6%, 4.7%, 4.7%, 11.6%, and 48.9% of patients, respectively. The double‐tract method was good, with less stenosis and reflux; however, the amount of residual food is noticeable. In addition, there are some cases in which contrast agent or diet do not pass to the remnant stomach. Therefore, comparison with TG will be of interest. The KLASS‐05 trial in Korea compares laparoscopic PG with double‐tract reconstruction and laparoscopic TG. The primary endpoint is the change in hemoglobin levels at two years after gastrectomy, and the secondary endpoints are the incidence rates of postoperative reflux esophagitis and anastomotic stricture, incidence of morbidity and mortality, QOL at 2‐year postgastrectomy, and three‐year disease‐free survival. The results of this study are awaited.

### 4.6 Jejunal interposition

The success of the jejunum interposition reconstruction method was announced to the world first by Prof. Merendino, who reported an animal experiment and its clinical application in jejunal interposition after PG.^46^

Eleven studies have reported results of jejunal interposition, with morbidity, stenosis, reflux esophagitis, and residual food occurring in 0%‐31.6%, 3.1%‐31.8%, 0%‐33.3%, and 8.5%‐31.8% of cases, respectively. ^5^, ^6^, ^30^, ^31^, ^32^, ^36^, ^39^, ^47^, ^48^, ^49^, ^50^

### 4.7 Jejunal pouch interposition

Residual food is the biggest problem in jejunal pouch reconstruction, and Nakamura et al. have reported food residues in >90% of cases.^36^ Several studies of patients who underwent a jejunal pouch interposition have reported the incidences of morbidity, stenosis, reflux esophagitis, and residual food as 3.6%‐25%, 0%‐27.8%, 4%‐27.8%, and 21.1%‐91.7%, respectively.^35^, ^36^, ^47^, ^49^, ^51^, ^52^

## 5 COMPARISON OF RECONSTRUCTION OUTCOMES AFTER PG

Comparisons between jejunal interposition and other reconstruction methods have been reported as follows: jejunal interposition vs esophagogastrostomy, n = 3^30^, ^32^, ^36^; jejunal interposition vs jejunal pouch interposition, n = 3^36^, ^47^, ^49^; and jejunal interposition vs tube‐like stomach esophagogastrostomy, n = 1.^39^ There was also a report comparing tube‐like stomach esophagogastrostomy with esophagogastrostomy (n = 1).^34^ Except for one study that compared jejunal pouch interposition and jejunal interposition in two randomized groups,^47^ most of the comparative studies were retrospective cohort studies.

Of the four retrospective studies comparing the outcomes of jejunal interposition and esophagogastrostomy, one study found increased early postoperative complications of jejunal interposition (20.0% vs 3.1%),^36^ two studies found a decreased risk of developing reflux esophagitis (0% vs 21.8% and 5.0% vs 32.4%, respectively),^32^, ^36^ and no significant differences in stenosis or emptying dysfunction between the two different methods were observed.

Of the three studies comparing the outcomes of jejunal interposition and jejunal pouch interposition, one retrospective study found an increased risk of reflux esophagitis in the jejunal interposition group (33.3% vs 11.3%),^49^ and another study found an increased incidence of residual food in the jejunal pouch interposition group (31.8% vs 91.7%).^36^ The only prospective, randomized study found an increased risk of early postoperative complications in the jejunal interposition group (31.6% vs. 5.3%).^47^

One retrospective study reported the outcomes of jejunal interposition and tube‐like stomach esophagogastrostomy.^39^ No significant differences in early complications, stenosis, or reflux esophagitis were found between the two groups.

In the one retrospective study comparing the outcomes of tube‐like stomach esophagogastrostomy and esophagogastrostomy, the tube‐like stomach procedure showed a decreased incidence of reflux esophagitis (5.7% vs 22.0%) and a similar incidence of stenosis and emptying dysfunction.^34^

With respect to the nutritional status, although a large retrospective study using the postgastrectomy syndrome assessment scale reported that PG reduces symptoms after gastrectomy syndrome more than TG,^4^ accurate comparison of nutritional status by the reconstructive procedure after PG would be quite difficult due to the large bias in the selection of the reconstructive procedure. Nakamura et al reported that EG significantly reduced weight loss 3 years after PG compared with reconstruction of esophagogastrostomy, jejunal interposition, and jejunal pouch interposition. Similarly, Sakuramoto et al^27^ also reported that the weight loss rate after 1 year was mild in esophagogastrostomy, although it was not significant in the comparison with reconstruction of esophagogastrostomy and double tract. Esophagogastrostomy after PG is a physiological reconstruction method and may have advantages in maintaining nutritional status.

## 6 GASTRIC CANCER IN THE REMNANT STOMACH AFTER PG

An important consideration in PG is the increased risk for remnant gastric cancer. The rate of remnant gastric cancer has been reported as higher after PG (3.6%‐9.1%) than after distal gastrectomy (0.4%‐2.5%). ^50^, ^53^, ^54^, ^55^ Aggressive endoscopic screening in asymptomatic patients leads to early detection and curative resection of gastric cancer in the remnant stomach.^56^, ^57^, ^58^ Although endoscopy intubation after esophagogastrostomy is not difficult, it can be a challenging procedure after esophagojejunostomy, especially in patients with a longer interposed segment.^32^, ^59^ Because the evaluation of the remnant stomach in patients with an interposed jejunum >10 cm long remains challenging, surgeons performing PG with jejunal interposition reconstruction should pay close attention to the length of the interposed jejunum when considering an endoscopic follow‐up.

## 7 CONCLUSIONS

Regarding the reconstruction method after PG, the characteristics of each surgical procedure should be understood so that an appropriate reconstruction method, with a reflux prevention mechanism and minimal postoperative injury, can be selected.

## Disclosure

Conflict of interests: There are no conflicts of interest to disclose.

Author Contributions*:* (I) conception and design: S Nunobe; (II) administrative support: S Nunobe; (III) provision of study materials or patients: all authors; (IV) collection and assembly of data: S Nunobe; (V) data analysis and interpretation: S Nunobe; (VI) manuscript writing: all authors; (VII) final approval of manuscript: all authors.
